# High-Quality Draft Genome Sequence of Actinobacterium Kibdelosporangium sp. MJ126-NF4, Producer of Type II Polyketide Azicemicins, Using Illumina and PacBio Technologies

**DOI:** 10.1128/genomeA.00114-15

**Published:** 2015-04-02

**Authors:** Yasushi Ogasawara, Norah Torrez-Martinez, Anthony D. Aragon, Benjamin J. Yackley, Jessica A. Weber, Anitha Sundararajan, Thiruvarangan Ramaraj, Jeremy S. Edwards, Charles E. Melançon

**Affiliations:** a Department of Chemistry and Chemical Biology, University of New Mexico, Albuquerque, New Mexico, USA; b Department of Molecular Genetics and Microbiology, University of New Mexico Health Sciences Center, Albuquerque, New Mexico, USA; c Department of Biology, University of New Mexico, Albuquerque, New Mexico, USA; d National Center for Genome Resources (NCGR), Santa Fe, New Mexico, USA; e Department of Chemical and Biological Engineering, University of New Mexico, Albuquerque, New Mexico, USA; f Cancer Research and Treatment Center, University of New Mexico Health Sciences Center, Albuquerque, New Mexico, USA; g Center for Biomedical Engineering, University of New Mexico, Albuquerque, New Mexico, USA

## Abstract

Here, we report the high-quality draft genome sequence of actinobacterium Kibdelosporangium sp. MJ126-NF4, producer of the type II polyketide azicemicins, obtained using Illumina and PacBio sequencing technologies. The 11.75-Mbp genome contains >11,000 genes and 22 polyketide and nonribosomal peptide natural product gene clusters.

## GENOME ANNOUNCEMENT

Actinobacteria are an important source of natural products, which are structurally complex small molecules that possess diverse and often medicinally useful bioactivities (1). Natural products are also increasingly recognized as playing important ecological roles (2). As improvements are made in bioinformatic methods for natural product gene cluster identification and functional prediction (3) and in techniques and hosts for sequence-based gene cluster capture (4) and heterologous expression (5), high-quality actinobacterial genome sequence data are becoming increasingly useful as the genetic template for the native or heterologous production of natural products and engineered analogues.

Although an increasing number of actinobacterial genomes have recently been sequenced, and recent phylogenetics-guided sequencing projects (6) provide a more complete genomic picture of phylum *Actinobacteria*, many actinobacterial genera do not yet have a representative genome sequence. The genomes of organisms from these genera represent an untapped source of natural-product biosynthetic diversity (7). Thus, we are interested in obtaining high-quality draft genome sequences of organisms from actinobacterial genera lacking genome-sequenced representatives, including those from the genus Kibdelosporangium.

The genus Kibdelosporangium, which is in the suborder *Pseudonocardineae*, is one of the rarest actinobacterial genera, with only 12 unique full-length 16S rRNA sequences from cultured isolates currently available in the NCBI databank. In spite of their rarity, members of the genus Kibdelosporangium produce a number of natural products with antibacterial, anticancer, and antiviral activities, including the structurally unusual aziridine-containing type II polyketide azicemicins produced by Kibdelosporangium sp. MJ126-NF4 (8), whose genome sequence is reported here. This organism, which was initially misclassified as Amycolatopsis sp. MJ126-NF4, was isolated from a soil sample collected in Setagaya-ku, Tokyo Prefecture, Japan, in 1993 (9). In 2009, the azicemicin biosynthetic gene cluster was sequenced and annotated, and biochemical studies were carried out to investigate the biosynthetic origin of the aziridine moiety (10).

Genome sequencing was carried out using Illumina MiSeq (San Diego, CA) and Pacific Biosciences RS II (Menlo Park, CA) sequencing platforms. The MiSeq paired-end data (one lane, 216-bp average read length, ≈440× coverage) were assembled using MIRA (11) and polished using a custom script, resulting in a 74-contig draft assembly. The polished synthetic reads from this assembly were combined with PacBio long reads (one single-molecule real-time [SMRT] cell, ≈38× coverage) and merged using MIRA, resulting in an improved 41-contig assembly. This assembly was further scaffolded with the same PacBio long reads using the A Hybrid Assembler (AHA) protocol from the SMRT Analysis package (12). Finally, the GapCloser (version 1.12-r6) module from the SOAP suite (13) was used to attempt to resolve N-spacers introduced during the assembly scaffolding process, resulting in a high-quality 11.75-Mbp (21-scaffold; N_50_, 1.6 Mbp) draft genome assembly.

Gene prediction and annotation were carried out using RAST (14), incorporating the Glimmer (15) algorithm, and identified 10,999 putative protein-coding genes, 7 rRNAs, and 63 tRNAs. Twenty-two polyketide and nonribosomal peptide natural-product biosynthetic gene clusters, all of which were intact, including the error-free azicemicin cluster, were identified using Dynamite (16) and confirmed using antiSMASH (17).

### Nucleotide sequence accession numbers.

This genome sequence has been deposited in EMBL/GenBank under the accession no. CDME00000000. The version described in this paper is the first version, CDME01000000.
